# Hot-Pressing Deformation Yields Fine-Grained, Highly Dense and (002) Textured Ru Targets

**DOI:** 10.3390/ma16206621

**Published:** 2023-10-10

**Authors:** Shaohong Liu, Fengshuo Xu, Limin Zhou, Hao Cui, Manmen Liu, Ming Wen, Chuanjun Wang, Wei Wang, Song Li, Xudong Sun

**Affiliations:** 1 Key Laboratory for Anisotropy and Texture of Materials (Ministry of Education), School of Materials Science and Engineering, Northeastern University, Shenyang 110819, China; 2 State Key Laboratory of Advanced Technologies for Comprehensive Utilization of Platinum Metals, Yunnan Precious Metals Laboratory Co., Ltd., Kunming 650106, China

**Keywords:** powder metallurgy, densification, grain preferential orientation, ruthenium, hot-pressing deformation

## Abstract

Ruthenium (Ru) is a refractory metal that has applications in the semiconductor industry as a sputtering target material. However, conventional powder metallurgy methods cannot produce dense and fine-grained Ru targets with preferred orientation. Here, we present a novel method of hot-pressing deformation to fabricate Ru targets with high relative density (98.8%), small grain size (~4.4 μm) and strong (002) texture. We demonstrate that applying pressures of 30–40 MPa at 1400 °C transforms cylindrical Ru samples into disk-shaped targets with nearly full densification in the central region. We also show that the hardness and the (002)/(101) peak intensity ratio of the targets increase with the pressure, indicating enhanced mechanical and crystallographic properties. Our study reveals the mechanisms of densification and texture formation of Ru targets by hot-pressing deformation.

## 1. Introduction

Ruthenium thin films are widely used in applications such as integrated circuits and magnetic recording materials [1,2,3,4,5]. These films are usually produced by magnetron sputtering of ruthenium targets, which has a significant influence on the film quality [2,4,5,6]. The target characteristics, such as density, grain size and texture, affect the sputtering rate and the film uniformity [2,5,7,8,9]. Therefore, it is desirable to fabricate ruthenium targets with fine grains, texture structure and full density.

The achievement of high density while having a fine microstructure in sintered refractory metals, such as Ruthenium (Ru), poses a formidable challenge because of the high temperature of sintering and the trouble in distinguishing between densification and the kinetics of grain growth [2,9,10,11]. To surmount these challenges, researchers have explored various techniques. These techniques include the utilization of field-assisted sintering methods, fine starting powders and grain growth inhibitors [9,10,11,12]. The use of fine/nanopowders has proven effective in suppressing grain size, thereby enhancing the hardness and strength of sintered refractory metals [9,10,11,12]. Grain growth inhibitors have been found to reduce surface energy and form stable second phases on the interface of inhibitors and sintered refractory metals [9,10,11,12]. This phenomenon slows down solution–reprecipitation reactions. Field-assisted sintering includes spark plasma sintering (SPS) and pulsed high-DC current sintering [9,10,11,12]. These techniques exhibit promise in achieving full density while preserving a fine microstructure in sintered refractory metals like Ru.

Refractory metal targets are commonly produced using powder metallurgy techniques. This manufacturing process can be categorized into two methods: “top-down” and “bottom-up” [9,10,11,13]. The “top-down” approach to fabricating refractory metal targets relies on the principle of severe plastic deformation. Typical severe plastic deformation, including equal-channel angular pressing and surface mechanical attrition, is employed to subject bulk metals to intense plastic deformation [9,12,14,15,16,17,18,19]. These methods are effective for refining the target microstructure and enhancing its performance. However, they are not suitable for refractory metals with low ductility [9]. In contrast, the “bottom-up” method involves synthesizing fine powders of refractory metals and sintering them under high temperatures [9,20,21,22]. This process allows for the production of dense and homogeneous refractory metal targets. However, it can also lead to the formation of residual pores and coarse grains, due to thermally driven densification and grain growth [9].

Ruthenium, a brittle and hard-to-work metal, presents challenges in achieving high density through severe plastic deformation [2,23,24]. Additionally, ruthenium requires ultra-high purity as a semiconductor sputtering target, which precludes the use of additives such as sintering aids and grain growth inhibitors [2,8,25]. Consequently, ruthenium targets can only be fabricated using the “bottom-up” method. This method is widely regarded as the most promising approach for the preparation of ruthenium targets due to its exceptional versatility in controlling the microstructure and its ability to fabricate samples with intricate geometries that achieve near-net-shape [9,10,11,12]. The “bottom-up” method commences with the utilization of fine starting powders. These powders are then compacted to form green bodies, which are subsequently subjected to high-temperature sintering. A major challenge for the “bottom-up” method is to strike a balance between two interrelated processes during sintering: grain growth and reduction of porosity (densification) [9,10,11,12]. The densification and grain growth phenomena are both driven by capillary forces, and their thermally activated kinetics often exhibit similar activation energies, making it challenging to independently regulate them. The similarity in their activation energies implies that the same thermal treatment can affect both phenomena simultaneously [9,10,11,12]. The preparation of ruthenium targets necessitates the use of extremely high sintering temperatures due to their high melting point, which further intensifies this concern. The application of elevated sintering temperatures can readily induce rapid coarsening of the grains, specifically referred to as abnormal grain growth, which simultaneously diminishes the density of sintered Ru targets [9,10,11,12]. In recent years, a plethora of studies have been conducted to address sintering challenges and achieve full-density textured ruthenium targets with refined microstructures [2,9,21,26,27,28,29]. The primary objective of these efforts is to enhance the density of Ru targets by employing special sintering approaches. These approaches encompass microwave sintering, spark plasma sintering (SPS), hot press (HP) sintering, hot isostatic press (HIP) sintering and resistance sintering under ultra-high pressure [2,9,21,26,27,28,29]. Microwaves, electric fields, external pressures and coupled multi-physical fields are employed to facilitate the sintering process, thereby achieving high density and a refined microstructure.

In this work, we adopt a new method to fabricate fine-grained and textured ruthenium targets, by applying hot-pressing deformation. This technique transformed cylindrical pre-sintered ruthenium bodies into disk-shaped targets with near-full densification in the central region. The targets’ density, hardness and (002) orientation increased with the deformation pressures. We achieved ruthenium targets with high relative density (98.8%), a small grain size (~4.4 μm) and strong (002) grain preferential orientation by hot-pressing deformation.

## 2. Experimental Section

### 2.1. Target Preparation

**Figure 1:**
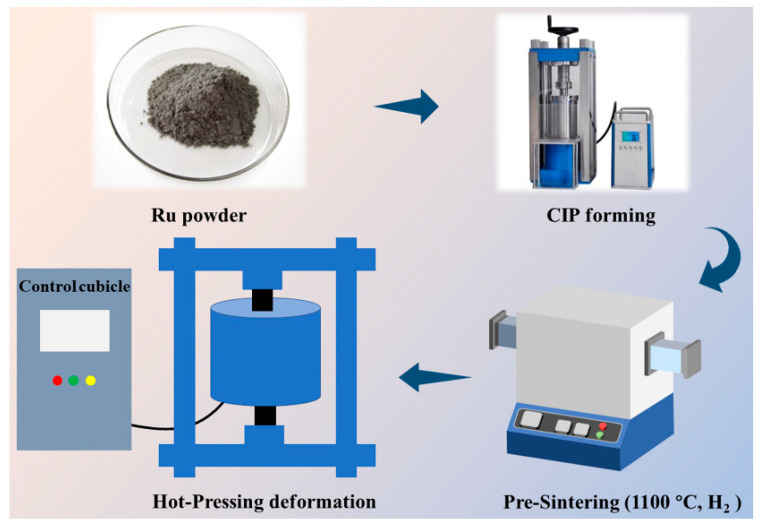
Schematic representation of the Ru target preparation process.

The fabrication of ruthenium (Ru) targets was carried out using commercially available Ru powder (purity 99.99%; Sino-Platinum Metals Co., Ltd., Kunming, China) as the starting powder. The Ru powder was subjected to cold isostatic pressing (CIP) at a pressure of 250 MPa in a 6 mm diameter polyurethane mold. This process led to the formation of green bodies. These green bodies were pre-sintered at a temperature of 1100 °C for 0.5 h under a hydrogen atmosphere. Eventually, the Ru targets were obtained by hot press sintering the pre-sintered bodies at pressures of 5, 10, 20, 30 and 40 MPa and a temperature of 1400 °C, under a vacuum of 10^−2^ Pa for 0.5 h in a graphite mold, which had an inner diameter of 15 mm. The Ru targets were subsequently ground and polished for performance analysis. The preparation process of the Ru target is illustrated in Figure 1 through a schematic diagram.

### 2.2. Characterization Techniques

The microstructure and morphology of Ruthenium (Ru) targets were analyzed using a field-emission scanning electron microscopy (FE-SEM), specifically Model JSM-7001F by Oxford Instruments HKL (Tokyo, Japan), operated at 15 kV. The Ru specimens were prepared following the standard metallographic sample preparation procedures. The corrosive liquid employed was a sodium hypochlorite solution. The phase composition of the Ru powder and targets was analyzed by X-ray diffraction (XRD) using a Panalytical PW 3040/60 (Eindhoven, The Netherlands) with nickel-filtered Cu Kα radiation and 40 kV and 200 mA operating conditions. The scanning range of 2θ is 30°–90° with a speed of 10° per minute. The density of the Ru targets was measured at room temperature using the Archimedes immersion method.

## 3. Results and Discussion

### 3.1. The Evolution of Appearance and Relative Density

**Figure 2:**
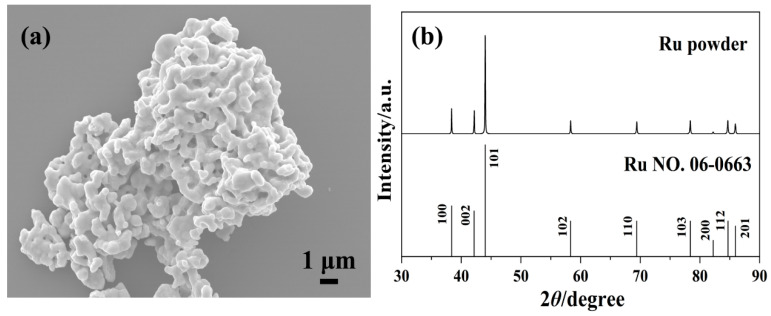
FE-SEM image and XRD pattern of the original Ru powder (**a**,**b**).

Figure 2 shows the FE-SEM image and XRD pattern of the original Ru powder. The commercial Ru powder with a high purity of 99.99% was used as the raw material. The powder has a fine particle size of approximately 500 nm and is severely agglomerated, with an agglomerate size of around 15 μm. The Ru particles are highly crystallized, and their X-ray diffraction pattern reveals the pure crystalline Ru phase (PDF#06-0663). No diffraction peaks of other phases are observed, except for the diffraction patterns of Ru particles.

**Figure 3:**
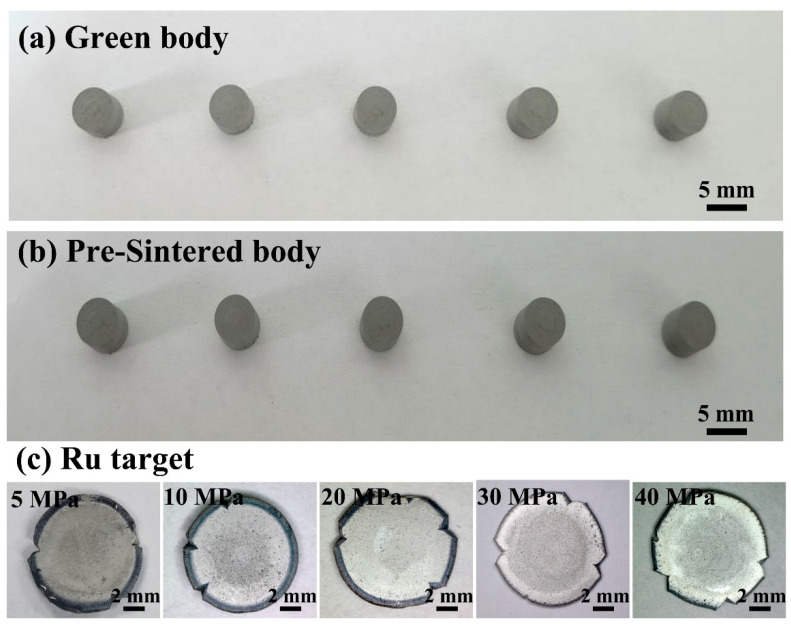
(**a**) Green bodies (cold isostatic pressing), (**b**) pre-sintered bodies (1100 °C, H_2_), and (**c**) Ru targets (hot-pressing deformation).

Figure 3 depicts the changes in the appearance of green bodies, pre-sintered bodies and Ru targets. The green bodies were obtained by cold isostatic pressing at 250 MPa in a polyurethane mold with an inside diameter of 6 mm. The green bodies exhibited a cylindrical shape and a dark gray color. The average diameter and height of green bodies were approximately 5.01 mm and 4.09 mm, respectively. After sintering the green bodies without pressure at 1100 °C for 0.5 h in a hydrogen atmosphere, pre-sintered bodies were obtained. The pre-sintered body also exhibited a dark gray color and retained the cylindrical shape. The average diameter and height of pre-sintered bodies were around 4.76 mm and 3.82 mm, respectively. As can be seen, after sintering without pressure in hydrogen at 1100 °C, the sample slightly densified, resulting in a slight reduction in the diameter and height.

The graphite mold used for hot-pressing deformation had an inner diameter of 15 mm, which was significantly larger than the diameter of the pre-sintered bodies. Therefore, during the hot-pressing sintering, deformation occurred under the applied hot-pressing pressure, due to the large difference between the sample diameter and the inside diameter of graphite mold. Hot-pressing deformation further densified the sample as a result of the simultaneous occurring of sintering and deformation. The resultant Ru target had a disk-like shape and showed a unique metallic luster due to high densification. When the hot-pressing pressure was increased from 5 MPa to 40 Mpa, the diameter of Ru target increased from 8.45 mm to 9.08 mm, while the height of Ru target declined from 1.04 mm to 0.76 mm. In addition, compared with the pre-sintered body, the diameter of the Ru target, deformed by hot pressing at 40 MPa, increased by 90%, while the height decreased by 80% (Table 1).

**Figure 4:**
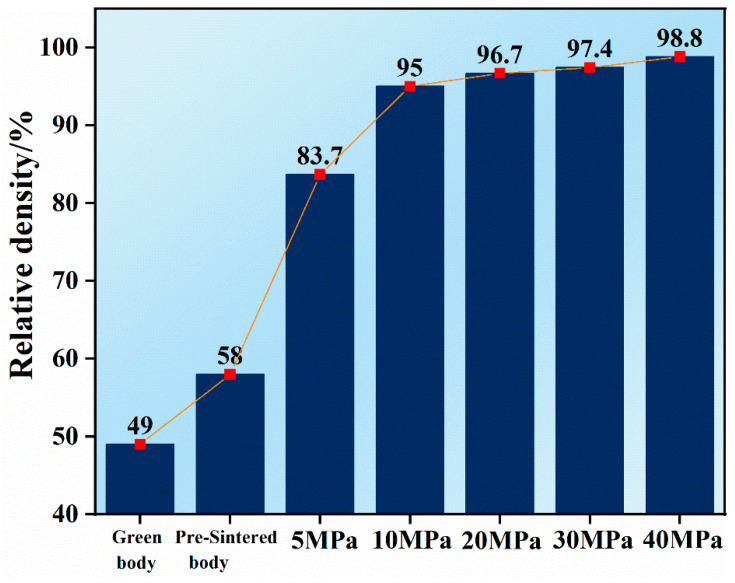
Relative density of green body (cold isostatic pressing), pre-sintered body (1100 °C, H_2_) and Ru targets following hot-pressing deformation at 5–40 MPa.

The relative density of samples significantly changed with the change in appearance, diameter and height. Figure 4 shows the relative density of green bodies, pre-sintered bodies and Ru targets following hot-pressing deformation at 5–40 MPa. The green body exhibited a relative density of 49%, whereas the pre-sintered body demonstrated a relative density of 58%. As the hot-pressing pressure increased from 5 MPa to 40 MPa, the relative density of the Ru target increased from 83.7% to 98.8%. The high relative density of up to 98.8% indicates high densification, which is the highest density of Ru target reported until now [2,5,27]. Therefore, hot-pressing deformation is a novel effective strategy for densifying refractory metals via powder metallurgy.

### 3.2. The Evolution of Microstructure and Hardness

Ruthenium is a refractory metal that is known for its high brittleness and poor workability, which makes it challenging to achieve full density by conventional powder metallurgy and severe plastic deformation (SPD) techniques [2,23,24]. Hot-pressing deformation could overcome these challenges, which involves sintering and deformation concurrently. Ru targets underwent hot-pressing deformation at 1400 °C and 10^−2^ Pa in a graphite mold for 0.5 h, with the hot-pressing pressure ranging from 5 MPa to 40 MPa. The high temperature of 1400 °C provides strong sintering driven force, while the pressure provides the deformation force to drive plastic deformation. The advantages of deformation include improving densification and grain refinement.

**Figure 5:**
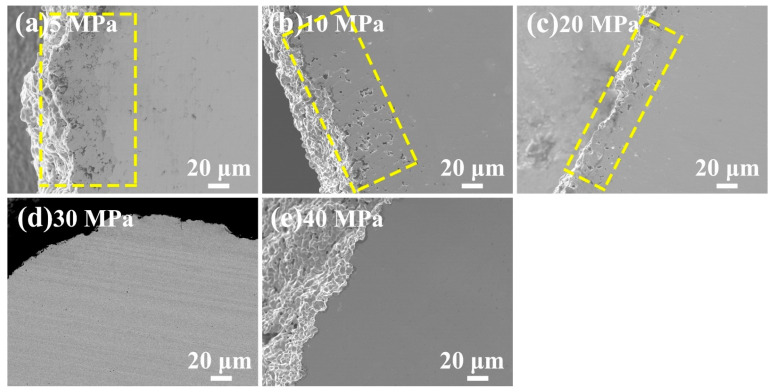
FE-SEM images showing the edge microstructure of Ru targets following hot-pressing deformation at 5–40 MPa.

The microstructure of the edge of Ru targets that underwent hot-pressing deformation at 5–40 MPa is presented in Figure 5. The edge region of Ru targets that underwent hot-pressing deformation at 5, 10 and 20 MPa exhibited numerous fine pores, indicating incomplete densification and lower density. The relative densities of these Ru targets were measured to be 83.7%, 95% and 96.7%, respectively, which were largely lower than the theoretical density due to the presence of numerous fine pores at the edge region. The depth of the edge region containing pores decreased with increasing hot-pressing pressure from 5 MPa to 20 MPa. When the hot-pressing pressure exceeded 30 MPa, the depth of the edge region containing larger pores approached zero. These results suggest that hot-pressing pressures below 20 MPa are insufficient to drive plastic deformation at 1400 °C. Higher hot-pressing pressures above 30 MPa are required to achieve rapid plastic deformation and densification. The density at the edge region increases with increasing hot-pressing pressure. As shown in Figure 5, Ru targets that underwent hot-pressing deformation at 40 MPa exhibit a very smooth and dense microstructure at the edge region.

**Figure 6:**
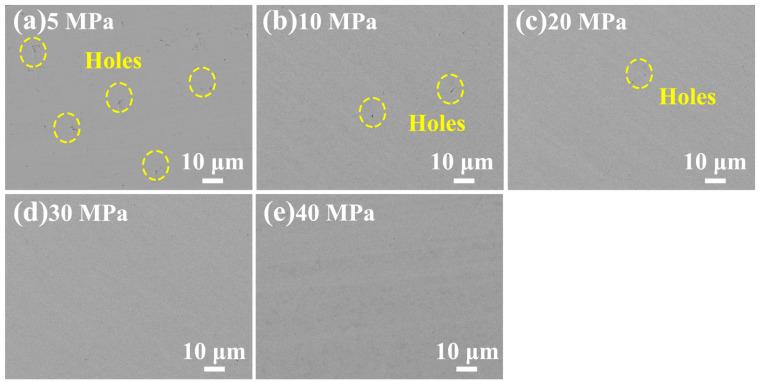
FE-SEM images showing the central microstructure of Ru targets following hot-pressing deformation at 5–40 MPa.

During the hot-pressing deformation process, cylindrical samples underwent a transformation from a cylindrical shape to a disk shape due to a decrease in height and an increase in diameter. This transformation resulted in the formation of distinct microstructures between the central and edge regions. The microstructure of the central region of Ru targets following hot-pressing deformation at 5–40 MPa is depicted in Figure 6. As compared with the edge microstructure shown in Figure 5, the central region of Ru targets exhibited a significantly denser microstructure than its edge region due to the hot-pressing deformation. However, the central region of Ru targets, following hot-pressing deformation at 5, 10 and 20 MPa, still contained small amounts of fine pores, indicating incomplete densification. The quantity of these fine pores in the central region of Ru targets decreased as the hot-pressing pressure increased from 5 MPa to 20 MPa, suggesting that increasing pressure improves densification. No pores were observed in the central region of Ru targets following hot-pressing deformation at 30 MPa and 40 MPa. This result indicates that when the hot-pressing pressure exceeded 30 MPa, the central region of Ru targets achieved nearly full density.

**Figure 7:**
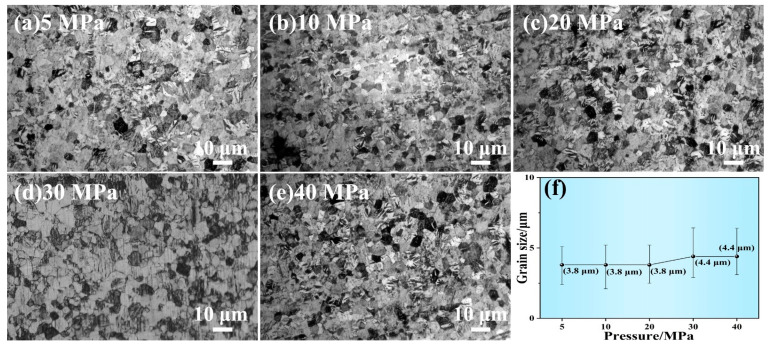
(**a**–**e**) The etched microstructure in the central region of Ru targets following hot-pressing deformation at 5–40 MPa, and (**f**) the average grain size as a function of the hot-pressing deformation pressure.

Hot-pressing deformation is a reliable technique for preventing rapid grain growth and achieving a refined microstructure. Figure 7a–e shows the etched microstructure in the central region of Ru targets that underwent hot-pressing deformation at pressures ranging from 5 MPa to 40 MPa. The microstructure of Ru targets following hot-pressing deformation at 5–40 MPa all exhibit a fine-grained structure, with no coarse or abnormal growth grains observed. Figure 7f demonstrates how the hot-pressing deformation pressure affects the average grain size of Ru targets. The average grain size of Ru targets following hot-pressing deformation at 5–20 MPa is around 3.8 μm, while the average grain size of Ru targets following hot-pressing deformation at 30–40 MPa is around 4.4 μm. As a result, fine-grained Ru targets are obtained.

Based on the results presented above, it is evident that hot-pressing deformation at pressures exceeding 30 MPa is a highly effective method for achieving dense Ru targets with a fine-grained microstructure. The Ru targets that undergo hot-pressing deformation at pressures above 30 MPa achieve a density of over 97% and fine grains of around 4.4 μm with a nearly fully dense central microstructure. These results provide valuable insights into the densification and grain refinement of Ru targets.

**Figure 8:**
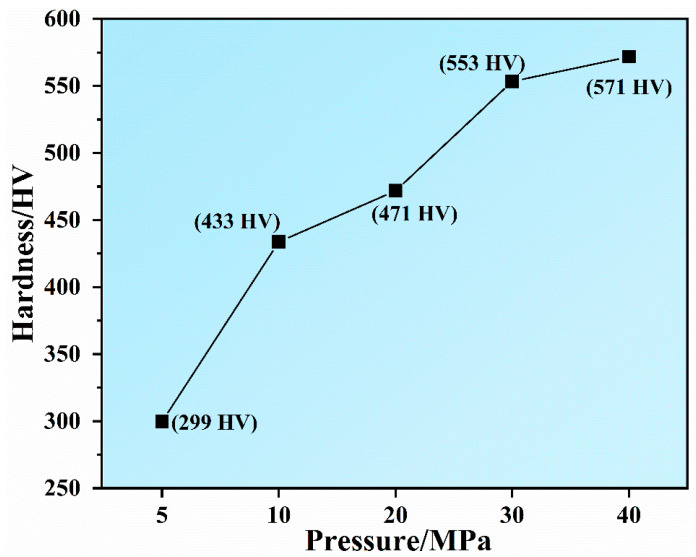
The Vickers hardness of Ru targets following hot-pressing deformation at 5–40 MPa.

The effect of hot-pressing deformation pressures on the hardness of Ru targets was investigated. Figure 8 shows the hardness of the Ru targets fabricated under various pressures from 5 MPa to 40 MPa. The average grain size of the Ru targets was almost close to each other for all pressures, implying that the grain size had no significant effect on the hardness variation. The main factor that influenced the hardness was the densification. The Ru target fabricated at 5 MPa exhibited a low hardness of only 299 HV due to the existence of more pores at the center and edge of the target. With increasing pressure, the pores were eliminated, and the overall densification improved, leading to a higher hardness. The Ru targets fabricated at pressures above 30 MPa reached a density of over 97% and a nearly fully dense central microstructure. Hence, the hardness of the Ru targets fabricated at 30 MPa and 40 MPa is as high as 553 HV and 571 HV, respectively, surpassing the previously reported data [2].

### 3.3. Grain Preferential Orientation

**Figure 9:**
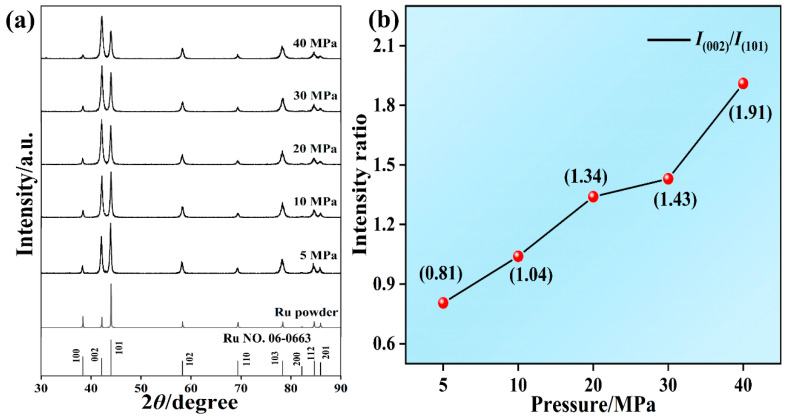
X-ray diffraction patterns (**a**) and the (002)/(101) peak intensity ratios (**b**) of Ru targets following hot-pressing deformation at 5–40 MPa.

Textured microstructures can be induced in Ru targets by hot-pressing deformation, which cannot be achieved by conventional powder metallurgy. The crystal structure of Ru targets remained unaffected by hot-pressing deformation, retaining the hexagonal structure (PDF#06-0663). However, Ru targets after hot-pressing deformation at 5–40 MPa exhibited a similar texture. The relative peak intensities were strongly affected by the hot-pressing deformation pressures, with the (002)/(101) peak intensity ratios significantly increasing with the increase in hot-pressing deformation pressures (Figure 9). This result means that the (002) grain preferential orientation occurred during hot-pressing deformation, and increasing the pressures could improve the (002) grain preferential orientation. The normal value of (002)/(101) peak intensity ratio is 0.35. However, the value of (002)/(101) peak intensity ratio of Ru targets fabricated at 40 MPa is as high as 1.91, indicating the strong (002) texture. The (002) grain preferential orientation is believed to be beneficial to improving the performance of Ru targets [2,5,8]. As a result, hot-pressing deformation not only achieved a nearly fully dense and fine-grained microstructure but also induced the strong (002) texture.

## 4. Conclusions

Fine-grained, highly dense and (002) textured Ru targets were fabricated by hot-pressing deformation at pressures above 30 MPa. The average grain size and relative density of the Ru targets were about 4.4 μm and over 97%, respectively. A nearly fully dense microstructure was observed in the central region of the targets. The hardness of the targets increased with the hot-pressing deformation pressure, reaching 553 HV and 571 HV at 30 MPa and 40 MPa, respectively. The (002) texture of the targets also enhanced with the hot-pressing deformation pressure, with a maximum (002)/(101) peak intensity ratio of 1.91 at 40 MPa. This study revealed new insights into the densification and texture formation of Ru targets and other refractory metals.

## Figures and Tables

**Table 1:** Diameter (D) and height (H) of samples.

Pressure (MPa)	Green Body		Pre-Sintering Body		Ru Target	
	D (mm)	H (mm)	D (mm)	H (mm)	D (mm)	H (mm)
5	4.98	4.04	4.77	3.86	8.45	1.04
10	4.98	4.12	4.74	3.87	8.71	0.88
20	5.07	4.02	4.82	3.76	9.01	0.82
30	5.00	4.13	4.76	3.79	9.03	0.78
40	5.01	4.14	4.72	3.83	9.08	0.76
